# Characterization of Visceral Adipose Tissue Proteome Reveals Metabolic Changes and Inflammatory Signatures in Severe Obesity

**DOI:** 10.1002/oby.70041

**Published:** 2025-10-12

**Authors:** Prince Dadson, Miikka‐Juhani Honka, Tomi Suomi, Anne Rokka, Saila Kauhanen, Paulina Salminen, Mika Helmiö, Peter James, Laura L. Elo, Vesa M. Olkkonen, Pirjo Nuutila

**Affiliations:** ^1^ Turku PET Centre University of Turku and Turku University Hospital Turku Finland; ^2^ Division of Information Science Nara Institute of Science and Technology Ikoma, Nara Japan; ^3^ Institute of Clinical Physiology National Research Council Pisa Italy; ^4^ Turku Bioscience Centre University of Turku and Åbo Akademi University Turku Finland; ^5^ Department of Surgery University of Turku Turku Finland; ^6^ Division of Digestive Surgery and Urology Turku University Hospital Turku Finland; ^7^ Department of Immunotechnology Lund University Lund Sweden; ^8^ Institute of Biomedicine University of Turku Turku Finland; ^9^ InFLAMES Research Flagship Center University of Turku Turku Finland; ^10^ Minerva Foundation Institute for Medical Research Helsinki Finland; ^11^ Department of Anatomy, Faculty of Medicine University of Helsinki Helsinki Finland; ^12^ Department of Endocrinology Turku University Hospital Turku Finland

**Keywords:** metabolomics, proteomics, severe obesity, visceral adipose tissue

## Abstract

**Objective:**

Severe obesity poses a major public health concern due to its links with cardiometabolic complications and mortality. Visceral adipose tissue (VAT) plays a key role in these processes through distinct molecular features. This study aimed to characterize the VAT proteome of individuals with severe obesity and investigate its association with serum metabolic biomarkers.

**Methods:**

A cross‐sectional analysis was performed for 46 individuals with severe obesity undergoing metabolic bariatric surgery and 17 healthy controls undergoing elective abdominal surgery. VAT proteomes were analyzed using liquid chromatography–tandem mass spectrometry (LC–MS/MS), and serum metabolites were quantified using nuclear magnetic resonance–based metabolomics.

**Results:**

LC–MS/MS identified 22 differentially expressed proteins (FDR < 0.05) in VAT with 12 downregulated and 10 upregulated in severe obesity. Downregulated proteins included mitochondrial enzymes involved in substrate metabolism and mitochondrial transmembrane transport. Circulating glucose, valine, and isoleucine correlated negatively with VAT mitochondrial transmembrane and electron transport proteins. Upregulated proteins were associated with inflammation, immune activation, oxidative stress, cytoskeletal remodeling, and protein turnover.

**Conclusions:**

These findings demonstrate significant molecular alterations in the VAT proteome associated with severe obesity, providing insights into the underlying mechanisms of metabolic disease. The differentially expressed proteins may serve as biomarkers or therapeutic targets for obesity‐related complications.

**Trial Registration:** ClinicalTrials.gov identifiers: NCT00793143 and NCT01373892

**Study Importance**

This study reveals key molecular changes in visceral adipose tissue of individuals with severe obesity. Using high‐throughput proteomics, we identified downregulation of proteins involved in lipid metabolism, mitochondrial function, and mRNA processing, alongside upregulation of inflammatory and immune‐related pathways. Many of the differentially expressed proteins have not been previously connected to obesity, or their role in adipose tissue is poorly understood. These findings provide a valuable resource for understanding the complex mechanisms driving severe obesity and the related metabolic dysfunction.

## 1 Introduction

Severe obesity, characterized by excessive adipose tissue accumulation and defined by a body mass index (BMI) ≥ 40 kg/m^2^, is a complex metabolic disorder and a leading global public health concern [1]. Adipose tissue is a complex and highly active metabolic and endocrine organ that plays crucial roles in regulating whole‐body energy homeostasis and metabolic processes [2]. It is distributed predominantly in the subcutaneous (SAT) and the visceral adipose tissue (VAT) depots, with differentiated structural, functional, and biochemical properties [2]. VAT is considered more metabolically active as it takes up higher amounts of glucose and fatty acids, cycles fatty acids more rapidly between lipolysis and reesterification, and has higher blood perfusion relative to its volume than SAT [3, 4]. VAT also has been shown to secrete increased levels of inflammatory cytokines [2]. Increased amounts of VAT, rather than the amount of SAT, have been associated with the development of several comorbidities such as hypertension, dyslipidemia, type 2 diabetes (T2DM), and cardiovascular diseases [2]. Despite extensive research, our understanding of the specific molecular components within the VAT depot and their intricate roles in metabolic dysregulation associated with severe obesity remains incomplete.

Developments in proteomics technology provide us with a powerful tool for global proteome analysis and identification of the differences between the adipose tissue depots [5]. High‐throughput proteomics techniques have enabled the reliable detection and quantitation of thousands of proteins in complex proteomes by liquid chromatography–tandem mass spectrometry (LC–MS/MS). Despite their enormous potential, there are very few high‐throughput proteomics studies comparing VAT in humans with obesity and its comorbidities to individuals without obesity. One study compared the VAT proteomes of women with severe obesity and with normal weight using high‐throughput label‐free one‐dimensional LC‐MS/MS [5]. Differentially expressed proteins in VAT were linked to the attenuation of the liver X receptor/retinoid X receptor pathway and the activation of the acute‐phase response [5]. In addition, a few older proteomics studies have compared VAT in people with and without obesity [6, 7, 8], but these studies used gel electrophoresis for protein separation which has low throughput because of lack of automation.

In this study, a high‐throughput LC–MS/MS quantification method was used to compare the protein abundances in VAT between patients with severe obesity and individuals without obesity as controls, with the goal of identifying proteins associated with cellular metabolic and signaling mediators in individuals with severe obesity. A combination of linear model analysis and gene set enrichment analysis (GSEA) was utilized to investigate the biological functions and pathways associated with the differentially expressed proteins. This study sheds light on potential mechanisms of adipose tissue dysfunction contributing to the pathogenesis of severe obesity. Furthermore, analysis of the VAT proteome provides a valuable resource and complement to existing knowledge, offering researchers a more comprehensive understanding of the molecular processes occurring within VAT in response to severe obesity or its associated comorbidities.

## 2 Methods

### 2.1 Design and General Characteristics of Study Participants

Datasets of two metabolic bariatric surgery imaging studies were combined for this study [3, 4]. A total of 46 patients with severe obesity (42 women and 4 men) were recruited from individuals undergoing metabolic bariatric surgery at the Hospital District of Southwest Finland. Inclusion criteria included participants aged 18–60 years with BMI ≥ 40 kg/m^2^ or ≥ 35 kg/m^2^ with an additional obesity‐related comorbidity. At baseline, the participants underwent clinical screening and imaging studies, and blood samples were collected for laboratory tests and serum metabolomics. After the baseline measurements, the participants had a 4‐week very low‐calorie diet (800 kcal/day) to reduce the risk of complications from the surgery due to excessive weight. During the diet, the participants lost approximately 7% of their body weight. A VAT sample was collected during the surgery. From these participants, a VAT sample was available for 39 individuals. In addition, VAT samples were obtained from 16 individuals without obesity (7 women and 9 men) undergoing elective abdominal surgical procedures for benign disorders, serving as controls to provide a comparative baseline for VAT analysis in the context of individuals with severe obesity. Before the surgery, the control participants underwent clinical examination, and blood samples were collected for laboratory tests and serum metabolomics. The controls did not follow a diet before the surgery, as this was not a requirement. Inclusion criteria for these controls were BMI ranging from 18 to 29.9 kg/m^2^, fasting plasma glucose levels < 6.1 mmol/L, and HbA1c levels < 6.1%. The study protocols were approved by the Ethics Committee of the Hospital District of Southwest Finland and conducted in accordance with the principles outlined in the Helsinki Declaration. The clinical trial registration numbers are NCT00793143 and NCT01373892. Details for the biochemical analyses, serum metabolomics, and adipocyte size measurements are provided in online Supporting Information File S1.

### 2.2 Preparation of VAT Samples

Biopsies from VAT were collected during bariatric surgery after a 4‐week very low‐calorie diet (800 kcal/day). VAT biopsies from metabolically healthy participants were collected during an elective intra‐abdominal surgery. The samples for proteomics were frozen in liquid nitrogen and stored at −70°C, and the samples for histology were stored in 10% formalin and embedded in paraffin.

### 2.3 Proteomics

#### 2.3.1 Sample Preparation

Adipose tissue samples were lysed, homogenized, and processed for protein extraction and quantification. High‐abundance serum proteins were depleted, and samples were prepared for LC–MS/MS analysis through in‐solution digestion and peptide desalting. Detailed protocols have been previously described [9] and are provided in online Supporting Information File S1.

#### 2.3.2 Liquid Chromatography–Tandem Mass Spectrometry Analysis

LC–MS/MS analyses were performed using a nanoflow HPLC system (Easy‐nLC1200, Thermo Fisher Scientific) coupled to a Q Exactive HF mass spectrometer with a nano‐electrospray ionization source, as previously described [9]. A detailed description is also provided in online Supporting Information File S1.

#### 2.3.3 Quantification

The R environment (version 4.0.4) was used for data analysis. Identifications to reverse peptide sequences and known contaminants were filtered from the data, and only proteins identified by two unique peptides were kept. This resulted in 2412 proteins from VAT. Proteins with an overall estimated intensity of zero were replaced by NA to denote missing values. The intensity matrix of all samples was normalized using variance stabilization normalization [10]. To filter adipose tissue samples that were contaminated with blood, a specialized scoring metric was used as previously described [9]. In brief, signature lists were acquired from the Human Protein Atlas (https://www.proteinatlas.org/) for adipose tissue (168 elevated genes) and blood (207 elevated genes). Calculating the score for each sample was done by first calculating the difference of protein intensities to their mean value over all samples, then performing a ranked GSEA [11] against the signature sets. The final score for each of the samples was defined as:

$$\begin{array}{c} \text{Score}=\\ \frac{\left[\left(1-p_{\text{adipose}}\right)\times Sign\left({\mathrm{ES}}_{\text{adipose}}\right)\right]-\left[\left(1-p_{\text{blood}}\right)\times Sign\left({\mathrm{ES}}_{\text{blood}}\right)\right]}{2} \end{array}$$

where ES is the enrichment score, and *p* is its significance. The final score, ranging from 1 (adipose tissue) to −1 (blood), represents the estimated bias. VAT samples with the highest blood‐like signature (below a cutoff of −0.75) were removed before further analyses were carried out (three samples from patients and three from controls).

### 2.4 Statistical Analysis

Differential expression between the sample groups was compared by using a linear model, where the potential confounding from weight loss before surgery, sex, and sleep apnea, as well as lipid and blood pressure medication, was accounted for. The potential effect of T2DM on the differential protein expression between the groups was also evaluated in an initial screen, but it did not show any effect and was therefore not included as a covariate in the final model. Benjamini–Hochberg procedure was used for multiple test correction. False discovery rate (FDR) < 0.05 was used as a threshold for differential expression to limit the proportion of false discoveries among the results presented and to focus on the protein findings that are most likely important. Spearman's rank correlation was used to estimate the relationships between clinical variables and protein abundances. It was calculated only if there were at least 10 measurements to compare. A ranked GSEA for the differential expression and correlation results was performed against the Molecular Signatures Database (MSigDB, version 2022.1.Hs) Hallmark and Gene Ontology (GO) sets using the R package fgsea. Protein–protein association networks based on a ranked list of the differential expression results were analyzed using Cytoscape stringApp v. 2.2.0. This analysis was performed using a minimum confidence score setting of 0.7, and the resulting large network was divided into smaller subnetworks by Markov clustering. The differences in the nuclear magnetic resonance (NMR) metabolomic measures between the groups with and without obesity were compared with logistic regression using the forest plot NMR R package and controlled for FDR (FDR < 0.05 was used as a threshold for significant findings).

## 3 Results

### 3.1 Anthropometric and Metabolic Characteristics of Participants With Obesity Versus Without Obesity

Individuals with severe obesity showed higher weight, BMI, waist circumference, body fat percentage, and larger adipocytes (Table 1). They also had elevated glucose metabolism markers, free fatty acids, and total cholesterol and lower HDL‐cholesterol, with no difference in triglycerides (Table 1).

**TABLE 1:** Anthropometric and metabolic characteristics of study participants.

Variables	Obesity (*n* = 46)	Controls (*n* = 17)	*p*
Age (years)	39.3 ± 16.3	43.1 ± 11.6	0.5323
Weight (kg)	116.7 ± 13.8	70.7 ± 10.5	< 0.0001
BMI (kg/m^2^)	41.3 ± 6.9	24.1 ± 2.7	< 0.0001
Waist circumference (cm)	91.2 ± 49.7	87.6 ± 8.0	< 0.0001
Body fat (%)	49.1 ± 5.8	27.2 ± 8.9	< 0.0001
Fat free mass (kg)	59.6 ± 11.1	43.2 ± 11.8	0.0001
Glucose (mmol/L)	6.3 ± 1.2	5.3 ± 0.4	0.0013
Insulin (μIU/mL)	14.4 ± 11.8	6.8 ± 3.5	0.0048
C‐peptide (nmol/L)	1.2 ± 0.4	0.6 ± 0.1	< 0.0001
HOMA‐IR	4.7 ± 5.4	1.6 ± 0.9	0.0278
Free fatty acids (mmol/L)	0.7 ± 0.22	0.41 ± 0.22	< 0.0001
Total cholesterol (mmol/L)	4.3 ± 0.8	5.0 ± 0.9	0.0037
Triglycerides (mmol/L)	1.25 ± 0.45	1.02 ± 0.44	0.0766
HDL cholesterol (mmol/L)	1.3 ± 0.2	1.7 ± 0.5	0.0001
LDL cholesterol (mmol/L)	2.5 ± 0.7	2.9 ± 0.8	0.0483
HbA1c (%)	6.0 ± 0.7	5.1 ± 0.3	< 0.0001
Visceral adipocyte size (μm)	91.5 ± 13.8	56.5 ± 11.1	< 0.0001

*Note*: Data are presented as mean ± SD. The Mann–Whitney *U* test was used for comparing individuals with obesity vs. controls. A *p* value < 0.05 was considered statistically significant. The metabolic biomarkers were measured at fasting.

Abbreviations: HbA1c: hemoglobin A1c; HDL: high‐density lipoprotein; HOMA‐IR: homeostatic model assessment of insulin resistance; LDL: low‐density lipoprotein cholesterol.

### 3.2 Serum Metabolomics Profiles in Participants With Obesity Versus Without Obesity

**FIGURE 1:**
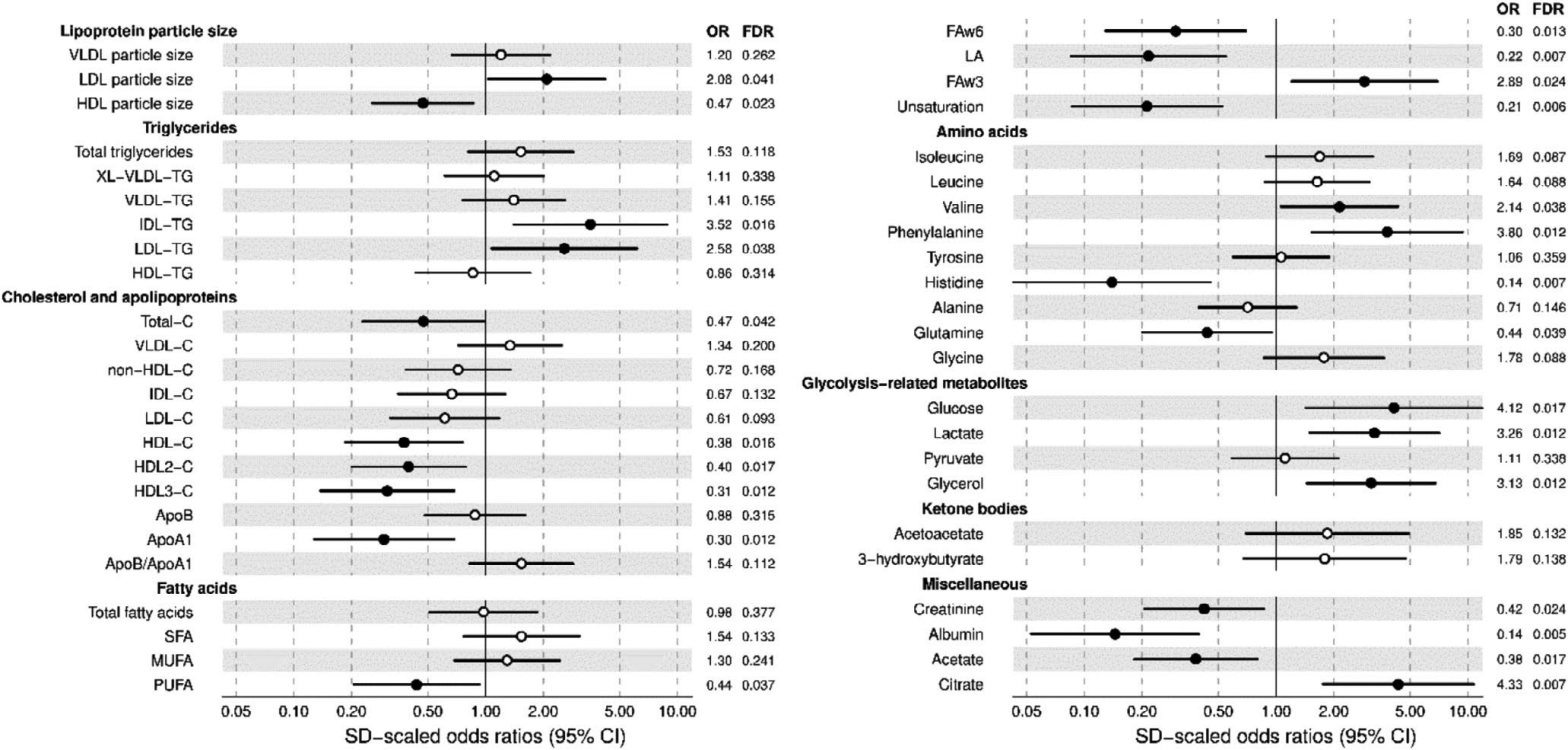
Cross‐sectional associations of metabolic measures with severe obesity. Odds ratios (OR) and their 95% confidence intervals are presented per 1 SD increment in the metabolic measures (log‐transformed) and shown with adjustment for age. Solid circles on the lines indicate FDR < 0.05. FAw3, omega‐3 fatty acid; FAw6, omega‐6 fatty acid; LA, linoleic acid; MUFA, monounsaturated fatty acid; PUFA, polyunsaturated fatty acid; SFA, saturated fatty acid.

Fifteen serum metabolic measures were downregulated and ten were upregulated in patients with severe obesity compared to individuals without obesity (FDR < 0.05). The participants with severe obesity had reduced HDL particle size, apolipoprotein A1, total cholesterol and HDL, HDL2, and HDL3 cholesterol, and degree of fatty acid unsaturation and increased LDL/IDL triglycerides and LDL diameter (odds ratios shown in Figure 1). Levels of omega‐6 and polyunsaturated fatty acids, linoleic acid, glutamine, histidine, creatinine, albumin, and acetate were decreased, while valine, phenylalanine, glucose, lactate, glycerol, citrate, and omega‐3 fatty acids were elevated (Figure 1).

### 3.3 Downregulation of Energy Metabolism, Amino Acid and Lipid Catabolism, and Cellular Signaling Pathways in Severe Obesity

**FIGURE 2:**
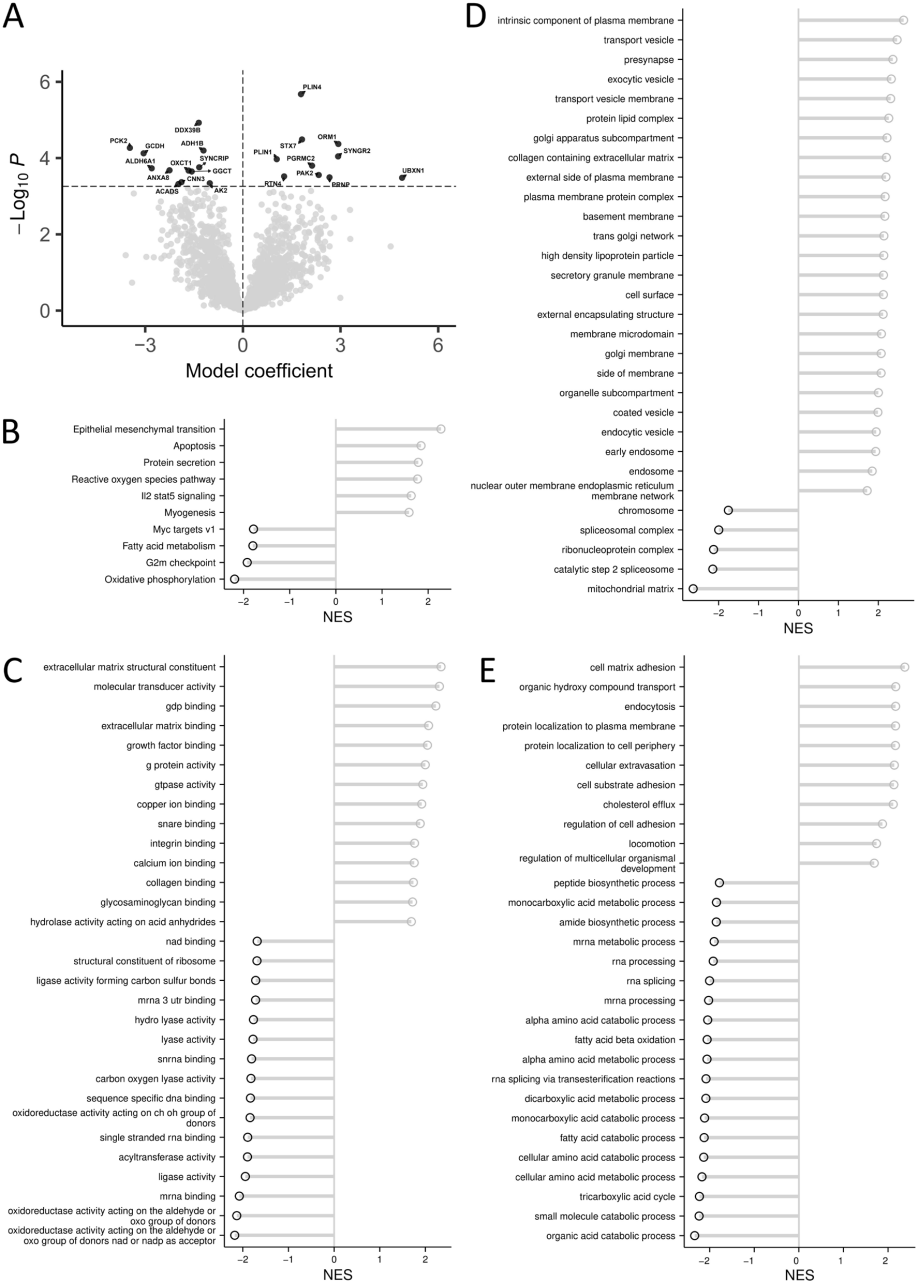
(A) Volcano plot describing the differences in expression of individual proteins in visceral adipose tissue (VAT) between patients with severe obesity and nonobese controls. The proteins with FDR < 0.05 in the comparison are highlighted with black. A positive coefficient means higher expression in the participants with severe obesity. Difference in (B) Molecular Signatures Database Hallmark, (C) gene ontology molecular function, (D) cellular component, and (E) biological pathway gene sets in VAT when comparing samples taken from patients with severe obesity and from nonobese controls. NES, normalized enrichment score. A larger NES indicates higher enrichment in obesity. Only gene sets with FDR < 0.05 are shown on the plots; max. 30 gene sets are shown in each plot.

**FIGURE 3:**
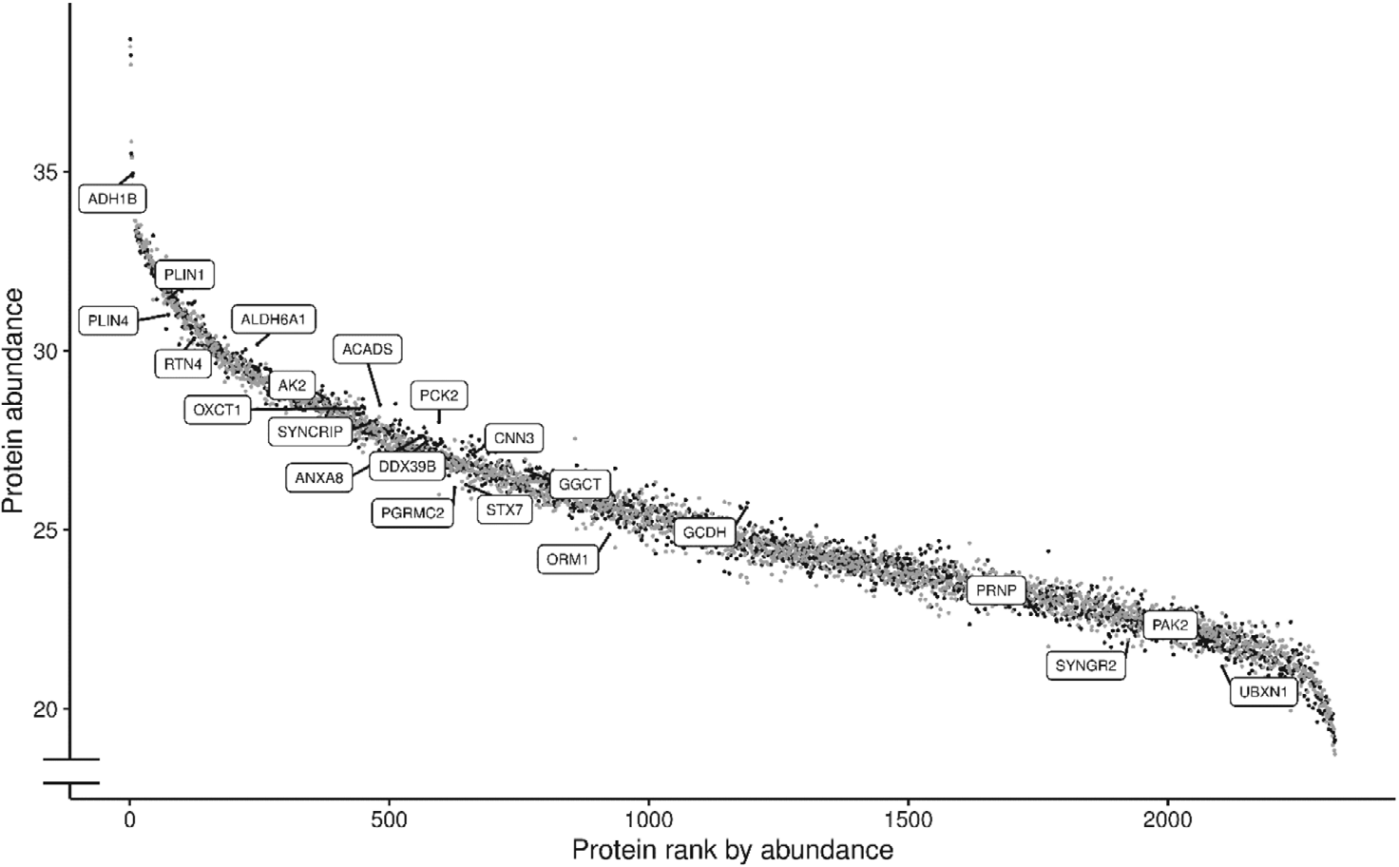
Visceral adipose tissue (VAT) protein abundance rank plot. In this plot the proteins are ranked highest to lowest by their mean normalized abundance. VAT protein abundance among nonobese controls is shown by black dots and in patients with severe obesity using gray dots. The most differentially expressed proteins between these groups (FDR < 0.05) are highlighted by the labels.

Twelve proteins were downregulated in severe obesity when accounting for false discovery rate (FDR < 0.05). These included several proteins involved in energy metabolism (e.g., AK2, ADH1B, ACADS, GCDH) (Figure 2A; online Supporting Information File S2), with ADH1B among the most abundant overall (Figure 3). Proteins linked to cellular signaling and stress responses, including SYNCRIP, DDX39B, CNN3, and ANXA8, were similarly reduced. Pathway enrichment analysis showed marked downregulation of oxidative phosphorylation, MYC targets, G2/M checkpoint regulation, and fatty acid metabolism in obesity (Figure 2B; online Supporting Information File S3). GO analysis further highlighted suppression of pathways related to amino acid and lipid catabolism, including branched‐chain amino acid (BCAA) metabolism, as well as aerobic respiration and the TCA cycle (Figures 2E and Figure S1A,B; online Supporting Information Files S2 and S4). Downregulated mitochondrial and transmembrane transport pathways were inversely associated with serum glucose, valine, and isoleucine levels (FDR < 0.05), while BCAA catabolism showed no such associations (FDR > 0.7) (online Supporting Information File S5). Apolipoprotein A1 was positively associated with catabolic enzymes, whereas apolipoprotein B showed inverse trends (FDR < 0.05) (online Supporting Information File S5). mRNA processing pathways (e.g., DDX39B, SYNCRIP, HNRNPs, PTBP1) were also downregulated (Figure S1D; online Supporting Information File S4), and mRNA processing pathway proteins correlating positively with serum glucose and apolipoproteins were enriched (FDR < 0.001; online Supporting Information File S5). Also, apolipoprotein A1 was strongly associated with HNRNPC, ‐R, and ‐U, while apolipoprotein B correlated with RALY (*r* > 0.5 for all, *p* < 0.01) (online Supporting Information File S6). Other processes downregulated in VAT in obesity included peptide biosynthesis, cytoplasmic translation, and sulfur metabolism. Despite the suppression of catabolic pathways, electron transport chain proteins remained largely unchanged (online Supporting Information File S4; Figure S1C).

### 3.4 Upregulation of Immune Response, Cellular Remodeling, and Metabolic Adaptations in Severe Obesity

Ten proteins were upregulated in severe obesity when accounting for false discovery rate (FDR < 0.05). Proteins involved in ubiquitination, lipid droplet formation and dynamics, and cytoskeletal remodeling were upregulated in individuals with severe obesity. These proteins were UBXN1, ORM1, SYNGR2, PRNP, PAK2, PGRMC2, STX7, PLIN4, RTN4, and PLIN1 (Figure 2A; online Supporting Information File S2). Pathway analysis showed a significant increase in the expression of proteins associated with epithelial‐mesenchymal transition (the most differentially expressed proteins in this pathway based on their rank included proteins such as CD44, FSTL1, LGALS1), apoptosis (e.g., CD44, GPX4, IGFBP6), protein secretion (e.g., STX7, M6PR, EGFR, RAB5A), reactive oxygen species regulation (e.g., PRNP, GLRX, GPX4), myogenesis (e.g., SYNGR2, PRNP, SORBS1, CRYAB), and IL2‐STAT5 (Figure 2B; online Supporting Information File S3). GO enrichment analysis revealed significant alterations in several biological processes associated with severe obesity. Compared to controls, immune‐related pathways such as adaptive immunity and leukocyte‐mediated processes (e.g., STX7, HPX, LAMP1, C5) were upregulated in obesity (Figure 2E; online Supporting Information File S4). Furthermore, vascularization and cell interaction pathways were upregulated, including blood vessel morphogenesis (C5, RRAS, SPARC, YAP1), cell adhesion, and extracellular matrix interactions (e.g., CD44, SORBS1, RRAS, LYVE1, ITGB1, LGALS1, CD248) (Figure 2E; online Supporting Information File S4). Additionally, a number of cell signaling and transport pathways were upregulated, involving processes such as exocytosis (e.g., SYNGR2, RAB11B, S100A10), Golgi organization (e.g., RAB1A‐B, RAB2B), ion transport (e.g., PGRMC2, PRNP, TF, RAB11B), neurotransmitter regulation (e.g., SNCG, ITGB1, RAB5A), and lipid transport (e.g., CES1, LAMTOR1, PLTP) (Figure 2E; online Supporting Information File S4). There were increased expressions of structural and functional pathways related to cell morphogenesis (e.g., PAK2, RTN4, CD44), locomotion (e.g., RTN4, TF, C5), and integrin‐mediated signaling (e.g., ITGB1, NRP1). Furthermore, protein modification and complex assembly pathways, including peptidyl tyrosine modification (e.g., PAK2, PRNP, CD44, HPX) and protein‐lipid complex organization (e.g., SAA4, PCYOX1, BIN1, PLTP), were upregulated. The cholesterol efflux pathway (e.g., CES1, LAMTOR1, PLTP) was upregulated, reflecting potential metabolic adaptations, while there was no difference in the fatty acid transport pathway (Figure 2E; online Supporting Information File S4).

## 4 Discussion

In the present study, we focused on differentially expressed proteins in VAT between individuals with severe obesity and individuals without obesity, in order to extend the understanding of the protein networks responsible for obesity and associated metabolic comorbidities. LC–MS/MS identified 22 differentially expressed proteins in VAT from the individuals with severe obesity compared to the individuals without obesity (FDR < 0.05). The downregulated proteins were involved in carbohydrate, lipid, and amino acid metabolism and energy production, consistent with a major remodeling of adipose tissue metabolic processes in obesity. In contrast, the upregulated proteins participated in immune response, inflammation, immune activation, cellular stress response, and protein turnover. It is of note that while the differentially expressed proteins correlated with visceral adipocyte size, BMI, and waist circumference in the whole population, there was a lack of association within the groups of severe obesity or controls for most of the proteins (online Supporting Information File S7). This suggests that, in these cases, the measures of adiposity were not directly associated with protein abundance but possibly through the previously mentioned processes.

### 4.1 Downregulation of Proteins Related to Lipid Metabolism, Mitochondrial Function, and mRNA Processing in Obesity

The current study found the downregulation of proteins crucial for fatty acid β‐oxidation (e.g., ACADS; a borderline difference for ACADM and HADH), suggesting impaired lipid metabolism in obesity, whereas there was no difference in the fatty acid transport pathway. ACADS is a mitochondrial protein that catalyzes the first step of the fatty acid β‐oxidation pathway. A previous study identified ACADS as an obesity‐related gene in humans, and adipose expression of ACADS was robustly correlated with BMI [12]. The downregulation of the fatty acid β‐oxidation pathway is compatible with our previous report showing unchanged fatty acid uptake per unit of VAT mass but reduced oxidative efficiency in obesity [3].

Proteins involved in energy metabolism, such as AK2, ADH1B, ACADS, and GCDH, were downregulated in patients with obesity, suggesting impaired lipid metabolism and mitochondrial dysfunction. Adenylate kinase 2 (AK2), a mitochondrial enzyme that regulates adenine nucleotide levels, has been linked to increased mitochondrial mass and oxidative phosphorylation [13], as well as adipocyte differentiation [14]. The latter study found that AK2 is a key enzyme for the induction of the unfolded protein response during the differentiation of 3T3‐L1 adipocytes, as well as in mature adipocytes, and its depletion led to reduced adiponectin secretion from the cells. In addition, the results suggested that the mechanism by which AK2 promotes the unfolded protein response is the supply of adenosine triphosphate to the endoplasmic reticulum. We have previously shown that AK2 expression is downregulated also in SAT in severe obesity [9]. Thus, the downregulation of AK2 may at least partly explain why adiponectin production is compromised in obesity [15]. Similarly, we found a decrease in the expression of ADH1B, an enzyme crucial for adipose tissue development and metabolic activity, which is known to be suppressed in obesity [16]. Given that ADH1B knockdown reduces insulin‐stimulated glucose uptake, its dysregulation in our study may reflect impaired insulin sensitivity [16], which is consistent with the reduced insulin‐stimulated VAT glucose uptake in obesity in our previous report [4]. Moreover, ANXA8 expression, previously reported to be similar in SAT and VAT of men with obesity [17], has been associated with cellular dysfunction when dysregulated [18]. Specifically, depletion of ANXA8 has been reported to cause accumulation of cholesterol into late endosomes and lysosomes where cholesterol levels are normally low [19]. Thus, although the function of ANXA8 is not well known, our findings suggest that its expression profile may play a role in alterations of VAT cholesterol metabolism in obesity. Consistent with previous observations, we also found downregulation of the acetyl‐CoA network in VAT and SAT, a hallmark of T2DM in individuals with obesity [20]. The reduced expression of genes such as ALDH6A1, ACAT1, and MTHFD1 in isolated adipocytes from diabetic individuals [21] aligns with our data, further supporting the metabolic consequences of impaired acetyl‐CoA metabolism in adipose tissue.

Heterogeneous nuclear ribonucleoproteins (HNRNPs) are a family of RNA‐binding proteins that play pivotal roles in the regulation of gene expression. HNRNP U has been implicated in the regulation of obesity‐associated meta‐inflammation and insulin resistance. In macrophages, HNRNP U translocates from the nucleus to the cytoplasm, where it stabilizes the mRNA of proinflammatory cytokines like TNFα and IL‐6. This stabilization contributes to the chronic low‐grade inflammation observed in obesity [22]. HNRNP K likely contributes to adipose tissue function by enhancing uncoupling protein‐2 (UCP‐2) mRNA stability and facilitating its mitochondrial translation, thereby enabling rapid modulation of UCP‐2 expression under metabolic stress [23]. In our study, the mRNA processing pathway was positively correlated with both apolipoprotein A1 and B. However, the spliceosome RNA helicase DDX39B, HNRNP Q (SYNCRIP), and HNRNPAK were significantly positively correlated only with apolipoprotein A1 but not B. DDX39B is a key enzyme involved in transcription, splicing, 3′‐end processing, and export of mRNA from the nucleus [24]. Interestingly, in contrast to the reduced expression in the current study, a previous study found DDX39B to be upregulated in VAT preadipocytes from people with obesity [25]. Because mature adipocytes are the commonest cell type in adipose tissue, the contrasting finding from these studies suggests that obesity affects the expression of DDX39B differently in preadipocytes and mature adipocytes.

The present results showed that amino acid and lipid catabolism were significantly downregulated in individuals with obesity. In a previous study, adipose tissue monomethyl BCAA content, linking BCAA metabolism to metabolic dysfunction, was lower in individuals with obesity compared with lean individuals [26]. Additionally, compatible with our results of the downregulated BCAA metabolic pathway in obesity, adipose tissue gene expression of enzymes involved in BCAA catabolism was reduced in insulin‐resistant mice and humans with obesity compared to their nonobese counterparts in a previous study [27].

In line with our findings, the study by Pérez‐Pérez et al. [7] identified downregulation in several other key proteins in fatty acid degradation such as ACADS, ACADM, HADH, and ACAT1 [7] in omental adipose tissue in obesity. Several of these enzymes are shared between lipid and amino acid catabolism. It is possible that the reduced BCAA catabolism in VAT could have contributed to the higher levels of circulating BCAAs observed in our study. However, the lack of correlation between the abundance of BCAA catabolic proteins in VAT and circulating BCAAs suggests that reduced BCAA catabolism in VAT is probably not a major contributor to the serum BCAA concentrations.

Several enzymes of the TCA cycle were found to be downregulated in VAT from the patients with severe obesity. Consistently, TCA cycle enzymes were in a previous study found downregulated in the SAT of individuals with acquired obesity [28]. Citrate produced in the citric acid cycle can be exported from mitochondria to cytosol via the transporter SLC25A1 [29], which was decreased in individuals with severe obesity in the present study. Consistently, previous proteomics analyses of SAT adipocytes of individuals with obesity and insulin resistance revealed a reduced abundance of SLC25A1 [30]. On the other hand, inhibition of SLC25A1 resulted in reduced weight gain and improved metabolic phenotype in mice fed a high‐fat diet [29].

### 4.2 Upregulation of Proteins Related to Inflammation, Immune Activation, Cellular Stress Response, and Protein Turnover

The results from GSEA showed an increase in inflammatory pathways in VAT from the patients with severe obesity. Consistently, in males with overweight/obesity and T2DM, VAT and VAT/SAT ratio were reported to be positively associated with CD163, a marker of macrophage activation [31].

The complement system is a key mediator of inflammation and may provide a link between adipose tissue inflammation and systemic metabolic derangements that promote human cardiometabolic disease [32]. Activation of the early pathways of the complement system results in the generation of C5a; together with C3a, C5a attracts cytokine‐producing macrophages and other leukocytes to the area of microbial invasion or tissue damage [33]. In contrast to our finding of increased C5 in VAT, the genes of the terminal pathway components C5 and C6 were downregulated in SAT and isolated adipocytes of heavier monozygotic twin pairs discordant for BMI [34]. This discrepancy may be due to tissue‐specific differences, as VAT is more metabolically active and has a stronger inflammatory profile compared to SAT [2, 3, 4]. Of note, syntaxin 7 (STX7), which was upregulated in our study, is a key component of a Q‐SNARE complex which facilitates matrix metalloproteinase MT1‐MMP trafficking in macrophages and the secretion of cytokines via the classical pathway [35]. Thus, while the role of STX7 in obesity has not been previously recognized, our results suggest that STX7 may contribute to the VAT inflammation in obesity.

Our results showed increased expression of CD44 in VAT in obesity. CD44 plays a crucial role in memory T cell development and migration, especially during inflammation [36]. In both mice and humans with obesity, CD44 expression was elevated in VAT, particularly in regulatory T cells (Tregs) and inflammatory macrophages. Of note, its reduction in the VAT Tregs correlated with an increase in CD11b+ CD11c+ macrophages [37]. It has been found that senescent CD4+ T cells, which produce high levels of osteopontin, promote macrophage migration, activate effector T cells, and suppress Treg function, leading to the maintenance of chronic inflammation in VAT [38].

In individuals with obesity, we found in the VAT an upregulation of PLIN1 and PLIN4, which belong to the class of perilipins, highly phosphorylated adipocyte proteins localized at the surface of lipid droplets [39]. A previous study found that both perilipin protein and mRNA levels were elevated in adipose tissue of people with obesity, potentially as a compensatory mechanism to reduce basal lipolysis [40]. However, no significant relationship was observed between perilipin levels and markers of insulin sensitivity in the current or the previous study [40].

Alpha‐1‐acid glycoprotein 1 (ORM1) was upregulated in our patients with severe obesity. A previous study has demonstrated that mice deficient in ORM1 display a phenotype associated with obesity, characterized by excessive collagen deposition in adipose tissue and enhanced expression of extracellular matrix regulators, including metalloproteinases and tissue inhibitors of metalloproteinases [41]. In another mice study, ORM1 suppressed proinflammatory gene expression, reactive oxygen species generation, and TNFα‐mediated lipolysis and improved glucose tolerance [42]. Thus, the upregulation of ORM1 in our patient population with obesity may represent a compensatory mechanism to prevent harmful effects of excess inflammation. The cholesterol efflux pathway was upregulated in VAT of individuals with severe obesity compared to controls in our study. In previous studies, the expression of CES1 has been found to be higher in large compared to small adipocytes from the same biopsy and upregulated in adipose tissue in individuals with obesity [5, 43]. Additionally, CES1 mRNA levels in adipose tissue were positively correlated with BMI, HOMA, fasting glucose, insulin, and triglyceride levels [44]. Based on in vitro and animal models, CES1 has been suggested to catalyze the hydrolysis of cholesteryl esters and triglycerides; however, knowledge regarding its function in human adipose tissue is scarce [45]. Our findings further showed an upregulation of LAMTOR1 and PLTP (phospholipid transfer protein) in severe obesity. Consistent with this observation, LAMTOR1 knockout mice showed in a previous study a decrease in body fat and a relatively smaller liver compared to the wild‐type mice [46]. Also, an overexpression of PLTP mRNA was observed in the omental fat in both men and women with obesity [17].

The present study has certain limitations. The participants with obesity had a 4‐week very low‐calorie diet before bariatric surgery as required to reduce the risk of complications, whereas the controls, who underwent elective surgery of the abdominal region, underwent no such presurgery diet. However, we used the percentage of weight change before surgery as a covariate in our analysis; the weight change did not have a major impact on our findings. This interpretation is also supported by the notion that our findings generally agree with what is previously known about the effects of obesity on VAT and with previous human VAT proteomics studies comparing individuals with and without obesity [5, 7, 8]. In addition, having the serum samples collected before the diet instead of on the surgery day in the group with severe obesity likely reduced our chances of observing more subtle associations between serum metabolites and VAT proteins. Furthermore, the group with severe obesity consisted of mostly women. However, we used sex as a covariate when comparing the groups with and without obesity, and sex had little effect on the analysis of differential protein expression. It is important to note that the study design was cross‐sectional; it thus identified associations and not definitive cause‐and‐effect relationships between the VAT proteome and metabolic health. Further research is needed to investigate these relationships longitudinally.

## 5 Conclusion

This study investigated the molecular underpinnings in the VAT of individuals with severe obesity at the proteomic level. We observed in these fat biopsies significant downregulation of proteins involved in fatty acid β‐oxidation, suggesting impaired lipid metabolism in individuals with obesity, potentially contributing to increased fat accumulation. Additionally, decreased expression of mitochondrial transporters and enzymes indicates compromised mitochondrial function. In contrast, proteins related to inflammation, immune activation, cellular stress response, and protein turnover were upregulated. These findings support an important role of chronic low‐grade inflammation in the pathologies associated with obesity. Overall, this study provides insights into the complex interplay between altered metabolism, mitochondrial dysfunction, and inflammatory processes in obesity. Lastly, our study identifies several differently regulated proteins whose role in obesity and adipose tissue is poorly known, such as ANXA8, DDX39B, STX7, SYNCRIP, SYNGR2, and PAK2. The present findings serve as a resource and pave the way for future research aimed at identifying new therapeutic targets for individuals with obesity and its comorbidities.

## Author Contributions

P.D., M.‐J.H., A.R., P.S., M.H., P.J., L.L.E., and P.N. conceived and designed the study. P.D., M.‐J.H., S.K., P.S., and M.H. collected tissue samples. A.R. and P.J. provided resources and expertise for the proteomics analysis. P.D., M.‐J.H., and T.S. analyzed data. P.D. and M.‐J.H. wrote the manuscript. V.M.O. interpreted the data and edited the manuscript. All authors critically revised the manuscript and approved the final version of the manuscript.

## Conflicts of Interest

The authors declare no conflicts of interest.

## Supporting information


**File S1:** Supplementary methods.

**File S2:** provides the result of the linear mixed effects models. Each sheet in the data includes Uniprot accession numbers (AC), corresponding gene names, and protein names, model coefficients (coef), *p* values, and false discovery rate (FDR). A positive coefficient for group on the sheet GROUPcontrol indicates higher level among the controls, whereas a negative coefficient means lower protein abundance (the groups were coded as 0 = patients; 2 = controls). The other sheets show the coefficients, *p* values, and FDR for the covariates: Presurgery_weightloss (%), LIPIDMEDyes (lipid medication; 0 = no; 1 = yes), BPMEDyes (hypertension medication; 0 = no; 1 = yes), SLEEPAPNEAyes (sleep apnea; 0 = no; 1 = yes), GENDERmale (0 = female; 1 = male).

**File S3:** Gene set enrichment analysis of Hallmark terms in patients with severe obesity before surgery and controls. Padj, false discovery rate (FDR)‐corrected *p* value; log2err, log2 transformed enrichment ratio; ES, enrichment score; NES, normalized enrichment score; size, number of proteins from the gene set included in the pathway analysis; leadingEdge, proteins that drive the enrichment. A positive ES and NES value in the table indicate an increased enrichment and a negative value decreased enrichment among patients compared to the controls.

**File S4:** Gene set enrichment analysis of Gene Ontology (GO) terms in patients with severe obesity before surgery and controls. GOCC, Gene Ontology Cellular Component; GOBP, Gene Ontology Biological Process; GOMF, Gene Ontology Molecular Function; padj, false discovery rate (FDR)‐corrected *p* value; log2err, log2 transformed enrichment ratio; ES, enrichment score; NES, normalized enrichment score; size, number of proteins from the gene set included in the pathway analysis; leadingEdge, proteins that drive the enrichment. A positive ES and NES value in the table indicate an increased enrichment and a negative value decreased enrichment among patients compared to the controls.

**File S5:** Gene Ontology (GO) enrichment analysis of proteins correlating with apolipoproteins A1 (ApoA1) and B (ApoB), fasting plasma glucose (FPG), isoleucine (Ile), and valine (Val). GOCC, Gene Ontology Cellular Component; GOBP, Gene Ontology Biological Process; GOMF, Gene Ontology Molecular Function; padj, false discovery rate (FDR)‐corrected *p* value; log2err, log2 transformed enrichment ratio; ES, enrichment score; NES, normalized enrichment score; Size, number of proteins from the gene set included in the pathway analysis.

**File S6:** Correlations of individual proteins with apolipoproteins A1 (ApoA1) and B (ApoB), fasting plasma glucose (FPG), isoleucine (Ile), valine (Val), and leucine (Leu).

**File S7:** Correlations of differently regulated proteins with visceral adipocyte size, BMI, and waist circumference.

**Figure S1:** Protein–protein network clusters for fatty acid and branched‐chain amino acid catabolism (A), tricarboxylic acid cycle (B), electron transport chain (C), and mRNA processing (D). Red color in the protein nodes indicates downregulation in obesity whereas blue means upregulation. Larger size and higher color intensity of the nodes indicate better confidence on the difference between the groups with or without obesity (based on the P value of group effect in the linear regression analysis). Width of the edges is based on confidence score for the interaction.

## Data Availability

The mass spectrometry proteomics data have been deposited to the ProteomeXchange Consortium via the PRIDE [47] partner repository with the dataset identifier PXD034182. The complete results for the differential expression analysis between the patients and controls, gene set enrichment, and correlation analyses are provided as online Supporting Information [Link], [Link]. Any additional information required to reanalyze the data reported in this paper is available from the corresponding author upon reasonable request.
