# Supplementary material for: Characterization of Visceral Adipose Tissue Proteome Reveals Metabolic Changes and Inflammatory Signatures in Severe Obesity
Source: Obesity (Silver Spring). 2025 Oct 12;34(1):127–37. doi: 10.1002/oby.70041 (PMC12724052; doi:10.1002/oby.70041)
Supplement: Supplementary file 1 — File S1: Supplementary methods. [file OBY-34-127-s003.docx]

**Dadson et al. Comprehensive Characterization of Visceral Adipose Tissue Proteome Reveals Distinct Metabolic Adaptations and Inflammatory Signatures in Severe Obesity**

**Supplementary methods**

**Biochemical and immunological analyses**

HbA1c (glycosylated haemoglobin) was measured using HPLC (Variant II; Bio-Rad) or Tina-quant Hemoglobin A1c Gen 3 assay (Cobas c501, Roche Diagnostics). Plasma insulin was assessed by ECLIA (Modular E170 or Cobas e601, Roche). Serum free fatty acids were determined using NEFA C enzymatic assay (Wako Chemicals) or Modular P800 (Roche Diagnostics). Plasma glucose was analyzed with Modular P800 or Cobas c702. C-peptide was measured via ECLIA on a Cobas e602 analyzer (Roche Diagnostics GmbH, Mannheim, Germany).

**Serum metabolomics with nuclear magnetic resonance (NMR) spectroscopy**

Serum metabolites were quantified using high-throughput proton NMR metabolomics (Nightingale Health Ltd, Helsinki, Finland; University of Eastern Finland, Kuopio, Finland) from fasting serum samples stored at -70°C (1). Briefly, before preparation, they were thawed overnight at +4 °C, gently mixed, and centrifuged at 3,400 × g to remove precipitate. Aliquots (300 µl) were combined with 300 µl of sodium phosphate buffer (75 mM Na₂HPO₄ in 80/20 H₂O/D₂O, pH 7.4) containing 0.08% TSP‑d₄ and 0.04% sodium azide. Sample preparation was automated using a Gilson Liquid Handler 215: buffer was first dispensed into 5 mm SampleJet NMR tubes, followed by serum, then mixed by three slow aspirations to prevent foam. Preparation of 96 samples took approximately two hours. A detailed description of this protocol is provided in Soininen et al., 2009 (2). This method allowed for the quantification of a broad range of metabolites, including total lipids, triglycerides, phospholipids, and various cholesterol components across different VLDL and HDL subfractions. Systemic lipid markers such as total cholesterol, triglycerides, and apolipoproteins A-I and B were also analyzed. Additionally, lipid constituents and fatty acid saturation diversity were measured from serum lipid extracts. Spectroscopy settings were optimized to detect small molecule solutes, including amino acids and glycolysis substrates, while suppressing broad spectral signals from lipoprotein particles (1).

**Adipocyte cell size measurements**

Digital images of hematoxylin-eosin-stained slides of the VAT samples were scanned with the Panoramic slide scanner system (v1.15.4; 3DHISTECH, Budapest, Hungary). Diameter of adipocytes (expressed in µm) of approximately 100 completely visible cells (3) within the scanned areas were manually outlined using ImageJ® (https://imagej.net/ij/). Values for the mean adipocyte size were computed from the measured adipocytes (4).

**Sample preparation**

Frozen adipose samples (~100 mg) were lysed using 500 µL of lysis buffer containing 1% sodium deoxycholate in 50 mM Tris-HCl (pH 8) supplemented with Complete EDTA-free protease inhibitor cocktail (Merck). Homogenization was performed using a TissueLyser LT instrument (QIAGEN) at +4°C with two 60-second cycles at 50 Hz, interspersed with a 5-minute cooling interval on ice. Subsequently, samples were incubated on ice for 30 minutes, followed by centrifugation at 16,000 ×g for 5 minutes at 4°C. The protein-containing supernatant, located beneath a lipid layer, was transferred to a new Eppendorf tube. Protein concentrations were quantified using a NanoDrop device (Thermo Scientific) by measuring absorbance at 280 nm.

To deplete high-abundance serum proteins from the adipose samples, Top12 Abundant Protein Depletion Spin Columns (Pierce Thermo, #85165) were utilized according to the manufacturer’s instructions, using 250 µg of total protein. Prior to and following the depletion, samples were concentrated using 10 kDa cut-off centrifugal filters (Microcon, MRCPRT010) to reduce volume. Protein concentrations in the depleted samples were determined using Bradford protein assay (BioRad). When feasible, 50 µg of each depleted sample was subjected to in-solution digestion. Proteins were reduced with dithiothreitol, alkylated with iodoacetamide, and digested with trypsin at a trypsin-to-protein ratio of 1:25. Sodium deoxycholate was removed prior to desalting by acidifying the digested samples and centrifuging at 16,000 ×g for 20 minutes. Desalting was carried out using a 96-well C18 plate (Waters) following the manufacturer’s protocol. Peptides were eluted with 0.1% formic acid in 50% acetonitrile. Prior to LC-MS/MS analysis, peptides were dissolved in 0.1% formic acid, and 500 ng of each sample was analyzed based on previously determined protein concentrations.

**Liquid Chromatography-Tandem Mass Spectrometry (LC-MS/MS) analysis**

The LC-MS/MS analyses were performed on a nanoflow HPLC system (Easy-nLC1200, ThermoFisher Scientific) coupled to a Q Exactive HF mass spectrometer (Thermo Fisher Scientific, Bremen, Germany) equipped with a nano-electrospray ionization source. Peptides were first loaded on a 2 cm trapping column and subsequently separated inline on a 15 cm C18 column (75 μm × 15 cm, ReproSil-Pur 5 μm 200 Å C18-AQ, Dr. Maisch HPLC GmbH, Ammerbuch-Entringen, Germany). The mobile phase consisted of water with 0.1% formic acid (solvent A) or acetonitrile/water (80:20 (v/v)) with 0.1% formic acid (solvent B). A 120 min chromatographic method was used to elute peptides (90 min gradient from 6% to 35% solvent B, following 8 min from 35% to 100% solvent B) followed by re-equilibration with A. A flow rate was 300 nl/min. MS data was acquired automatically by using Thermo Xcalibur 4.1 software (Thermo Fisher Scientific). A data dependent acquisition method consisted of an Orbitrap MS survey scan of mass range 300–2000 m/z followed by HCD fragmentation of the 10 most intense peptide ions in each scan cycle.

Raw MS files were processed using MaxQuant (version 1.6.5.0) (5) and searched against a UniProt/Swiss-Prot Homo sapiens database (4 May 2019, 20,418 entries). Matching between runs utilized a 0.7 min matching time window and a 20 min alignment time window. Methionine oxidation and N-terminal acetylation were set as variable modifications, while cysteine carbamidomethylation was fixed. Trypsin was used for protein digestion with allowance for up to two mis-cleavages. A false discovery rate (FDR) of 1% was applied for peptide and protein identifications. Protein quantification was based on the total identified peptide-ion intensities (MS).

**References**

1. Würtz P, Tiainen M, Mäkinen V-P, *et al.* Circulating Metabolite Predictors of Glycemia in Middle-Aged Men and Women. *Diabetes Care* 2012;35:1749–1756.

2. Soininen P, Kangas AJ, Würtz P, *et al.* High-throughput serum NMR metabonomics for cost-effective holistic studies on systemic metabolism. *Analyst* 2009;134:1781–1785.

3. Hoffstedt J, Andersson DP, Eriksson Hogling D, *et al.* Long-term Protective Changes in Adipose Tissue After Gastric Bypass. *Diabetes Care* 2017;40:77–84.

4. Tchoukalova YD, Koutsari C, Karpyak MV, Votruba SB, Wendland E, Jensen MD. Subcutaneous adipocyte size and body fat distribution. *Am J Clin Nutr* 2008;87:56–63.

5. Tyanova S, Temu T, Cox J. The MaxQuant computational platform for mass spectrometry-based shotgun proteomics. *Nat Protoc* 2016;11:2301–2319.
