# Supplementary material for: Characterization of Visceral Adipose Tissue Proteome Reveals Metabolic Changes and Inflammatory Signatures in Severe Obesity
Source: Obesity (Silver Spring). 2025 Oct 12;34(1):127–37. doi: 10.1002/oby.70041 (PMC12724052; doi:10.1002/oby.70041)
Supplement: Supplementary file 8 — Figure S1: Protein–protein network clusters for fatty acid and branched‐chain amino acid catabolism (A), tricarboxylic acid cycle (B), electron transport chain (C), and mRNA processing (D). Red color in the protein nodes indicates downregulation in obesity whereas blue means upregulation. Larger size and higher color intensity of the nodes indicate better confidence on the difference between the groups with or without obesity (based on the P value of group effect in the linear regression analysis). Width of the edges is based on confidence score for the interaction. [file OBY-34-127-s002.docx]

**
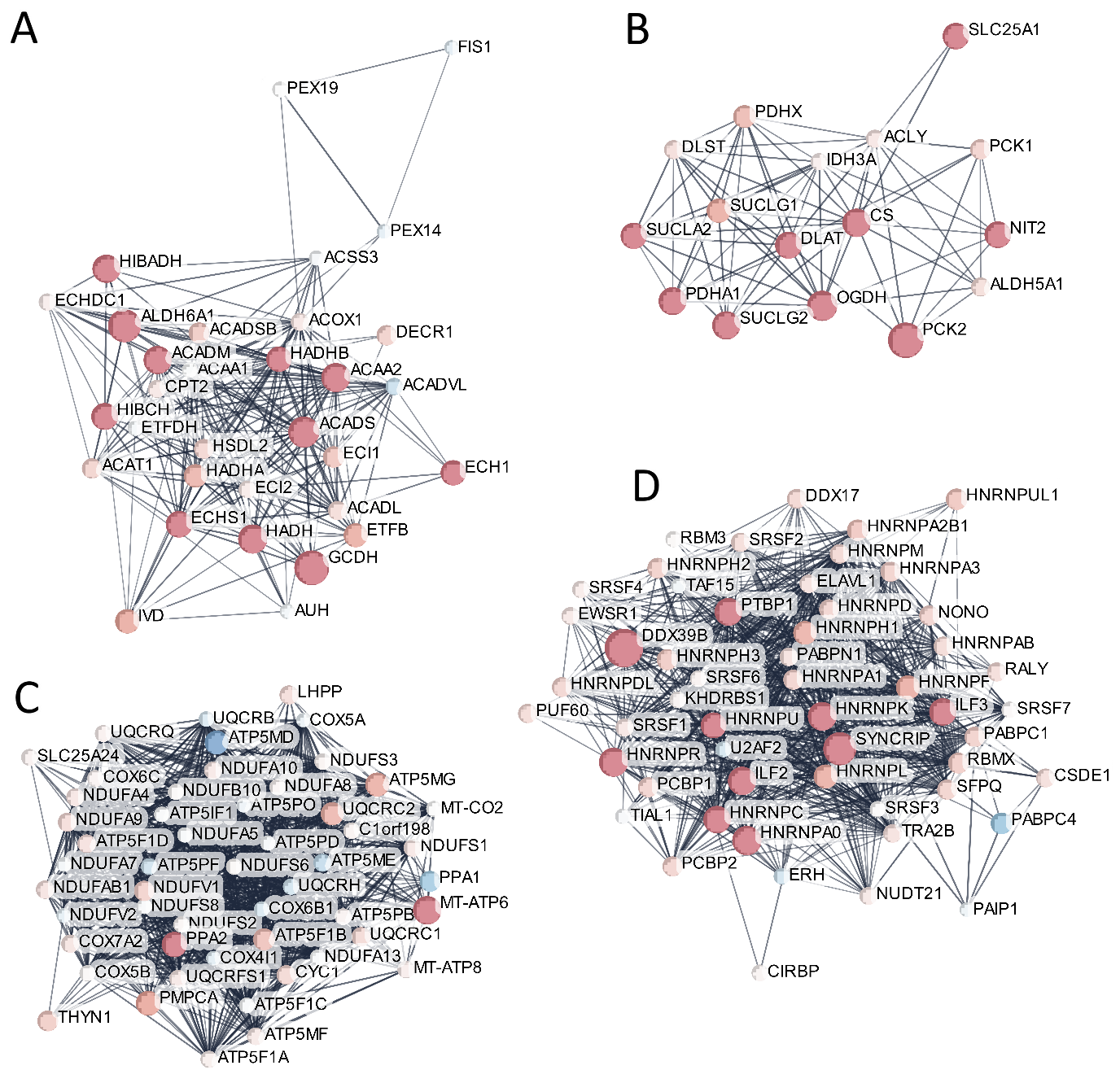
**

**Supplementary Figure S1.** Protein-protein network clusters for fatty acid and branched-chain amino acid catabolism (A), tricarboxylic acid cycle (B), electron transport chain (C), and mRNA processing (D). Red colour in the protein nodes indicates downregulation in obesity whereas blue means upregulation. Larger size and higher colour intensity of the nodes indicate better confidence on the difference between the groups with or without obesity (based on the P value of group effect in the linear regression analysis). Width of the edges is based on confidence score for the interaction.

**Supplementary File S1**. Supplementary methods.

**Supplementary File S2** provides the result of the linear mixed effects models. Each sheet in the data includes Uniprot accession numbers (AC), corresponding gene names, and protein names, model coefficients (coef), p-values, and false discovery rate (FDR). A positive coefficient for group on the sheet GROUPcontrol indicates higher level among the controls, whereas a negative coefficient means lower protein abundance (the groups were coded as 0=patients; 2=controls). The other sheets show the coefficients, p-values, and FDR for the covariates: Presurgery_weightloss (%), LIPIDMEDyes (lipid medication; 0=no; 1=yes), BPMEDyes (hypertension medication; 0=no; 1=yes), SLEEPAPNEAyes (sleep apnea; 0=no; 1=yes), GENDERmale (0=female; 1=male).

**Supplementary File S3.** Gene set enrichment analysis of Hallmark terms in patients with severe obesity before surgery and controls. Padj, false discovery rate (FDR)-corrected P-value; log2err, log2 transformed enrichment ratio; ES, enrichment score; NES, normalized enrichment score; size, number of proteins from the gene set included in the pathway analysis; leadingEdge, proteins that drive the enrichment. A positive ES and NES value in the table indicate an increased enrichment and a negative value decreased enrichment among patients compared to the controls

**Supplementary File S4.** Gene set enrichment analysis of Gene Ontology (GO) terms in patients with severe obesity before surgery and controls. GOCC, Gene Ontology Cellular Component; GOBP, Gene Ontology Biological Process; GOMF, Gene Ontology Molecular Function; padj, false discovery rate (FDR)-corrected P-value; log2err, log2 transformed enrichment ratio; ES, enrichment score; NES, normalized enrichment score; size, number of proteins from the gene set included in the pathway analysis; leadingEdge, proteins that drive the enrichment. A positive ES and NES value in the table indicate an increased enrichment and a negative value decreased enrichment among patients compared to the controls.

**Supplementary File S5.** Gene Ontology (GO) enrichment analysis of proteins correlating with apolipoproteins A1 (ApoA1) and B (ApoB), fasting plasma glucose (FPG), isoleucine (Ile), and valine (Val). GOCC, Gene Ontology Cellular Component; GOBP, Gene Ontology Biological Process; GOMF, Gene Ontology Molecular Function; padj, false discovery rate (FDR)-corrected P-value; log2err, log2 transformed enrichment ratio; ES, enrichment score; NES, normalized enrichment score; Size, number of proteins from the gene set included in the pathway analysis.

**Supplementary File S6.** Correlations of individual proteins with apolipoproteins A1 (ApoA1) and B (ApoB), fasting plasma glucose (FPG), isoleucine (Ile), valine (Val), and leucine (Leu).

**Supplementary File S7.** Correlations of differently regulated proteins with visceral adipocyte size, BMI, and waist circumference.
